# Maple Syrup: Chemical Analysis and Nutritional Profile, Health Impacts, Safety and Quality Control, and Food Industry Applications

**DOI:** 10.3390/ijerph192013684

**Published:** 2022-10-21

**Authors:** Ariana Saraiva, Conrado Carrascosa, Fernando Ramos, Dele Raheem, Maria Lopes, António Raposo

**Affiliations:** 1 Department of Animal Pathology and Production, Bromatology and Food Technology, Faculty of Veterinary, Universidad de Las Palmas de Gran Canaria, Trasmontaña s/n, 35413 Arucas, Spain; 2 Faculty of Pharmacy, University of Coimbra, Azinhaga de Santa Comba, 3000-548 Coimbra, Portugal; 3 Associated Laboratory for Green Chemistry (LAQV) of the Network of Chemistry and Technology (REQUIMTE), Rua D. Manuel II, Apartado 55142, 4051-401 Porto, Portugal; 4 Northern Institute for Environmental and Minority Law (NIEM), Arctic Centre, University of Lapland, 96101 Rovaniemi, Finland; 5 CBIOS (Research Center for Biosciences and Health Technologies), Universidade Lusófona de Humanidades e Tecnologias, Campo Grande 376, 1749-024 Lisboa, Portugal

**Keywords:** maple syrup, food industry, nutrition, chemical analysis, health impacts

## Abstract

Maple syrup is a delicacy prepared by boiling the sap taken from numerous Acer species, primarily sugar maple trees. Compared to other natural sweeteners, maple syrup is believed to be preferable to refined sugar for its high concentration of phenolic compounds and mineral content. The presence of organic acids (malic acid), amino acids and relevant amounts of minerals, such as potassium, calcium, zinc and manganese, make maple syrup unique. Given the growing demand for naturally derived sweeteners over the past decade, this review paper deals with and discusses in detail the most important aspects of chemical maple syrup analyses, with a particular emphasis on the advantages and disadvantages of the different analytical approaches. A successful utilization on the application of maple syrup in the food industry, will rely on a better understanding of its safety, quality control, nutritional profile, and health impacts, including its sustainability issues.

## 1. Introduction

Maple syrup is a delicacy prepared by boiling the sap taken from different Acer species, mainly sugar maple (*Acer saccharum* Marsh.) trees [1]. Agriculture and Agri-Food Canada [2] reports Canada as the world’s largest producer of maple products and it is responsible for nearly 71% of the maple syrup production in the world. In 2017, Quebec produced approximately 92% of all the maple syrup in Canada and is home to more than 13,300 maple syrup producers [3].

Of the many natural sweeteners, maple syrup is recognized as a much superior alternative to refined sugar for not only its mineral content, but also for its high concentration of phenolic compounds with bioactivity properties, i.e., anti-mutagenic, anti-radical, antioxidant, and anti-cancer [4,5,6]. Compared to dextrose, corn syrup and brown rice syrup, maple syrup brings about lower glucose and insulin responses, which make it a healthier substitute for refined sugars in our diet [4,7].

Maple tapping often begins late in winter or early in spring. It only lasts a few weeks because of the weather. To make maple syrup, sweet watery xylem sap is collected and concentrated. As a result of the pressure build-up caused by the freeze-thaw cycle, this sap pours out of maple tree trunks. To make one liter of maple syrup, around 40 liters of sap (containing 2–3% sugar) are required (66% sugar). Other than sucrose, which is maple syrup’s principal sugar, its flavor is a complex mix of not only minerals, amino acids, oligosaccharides, organic acids, and phenolic and volatile aromatic compounds, but also microbial contaminants from maple sap [8,9]. According to Filteau et al. [9], as well as its associated microflora, sap content can change during seasons and syrup color usually darkens as the season progresses.

As previously stated, the chemical composition of sap and syrup can significantly differ depending on geographical origin [10,11]. Indigenous peoples in North America introduced maple syrup to colonizing Europeans. Since then, maple syrup and maple products have been commercially sold [12]. More interest has been shown in studying the elemental composition of maple syrup as commercial markets expand and analytical technologies improve. As a result, several scientific research works have been conducted in the last century to determine the chemical components and mineral constitution of maple syrup [13].

According to these premises, the present review intends to examine the nutritional profile and health impacts of maple syrup consumption, some of its possible food industry applications and its sustainability issues, along with its main safety and quality parameters, and the chemical analysis of its principal components.

## 2. Chemical Analysis—Advantages and Disadvantages of the Different Analytical Methods

Maple syrup has a long-standing history of consumption, particularly in North America, where it is very much appreciated. More recently, interest in this product has spread to other areas of the globe like Europe and Japan, owing to the demand for natural sweeteners [14,15]. Maple syrup is regarded as a high-value product for its unique flavor [16]. Accordingly, diverse tools have been proposed to assess its quality and authenticity, protect consumers, and ensure fair competition among producers [17]. 

Maple syrup quality is assessed according to classification schedules in the USA and Canada, with typical standards applied to judge this product’s price, including the product’s color intensity (light-colored products tend to be more expensive). This means that syrup darkening can be used as an indicator of the irregularities that might arise during processing or microbial contamination [18]. Lighter maple syrups tend to be typically sweet and contain no further prominent flavors, while darker ones possess burned caramel flavors and are apt to be blended with light syrups for a more classic “maple flavor” [11]. Past consumer studies report a preference for darker maple syrups over lighter ones [19]. These darker syrups contain more beneficial bioactive compounds, such as polyphenols [20,21]. That being said, the Canadian Food Inspection Agency (CFIA) monitors Canadian maple syrup safety and quality [22] so that producers meet high federal standards. The CFIA is also responsible for the federal classification of Canadian maple syrup color descriptors and grades by ensuring that they align with standard international grading systems. Canadian regulation defines two grades (Grade A and Processing Grade) and four color classes (Gold, Amber, Dark, Very dark) for maple syrup.

Grade A Maple Syrup: according to the definitions dictated by the product specification, maple syrup can be classified as grade A, but only if it meets these requirements:

- With no undesired uniform fermentation color;
- Sediment-free. No turbidity;
- Characteristic natural maple flavor for all four color classes;
- No uncharacteristic odors or flavors.

Processing Grade Maple syrup: the maple syrup called processing grade is also obtained from maple sap concentration, but does not respect at least one of the quality parameters defined for Grade A or more.

To ensure its stability, maple sugar must not contain more than 10% moisture.

Grade A maple syrup can differ in the four color classes, defined by either a transmittance value or the ratio between the intensity of the light passing through samples and that of the light emerging from them [23]. The higher the transmittance value, the clearer and more transparent maple syrup is. The lower the transmittance value, the darker and denser maple syrup is.

It is nature itself that characterizes maple syrup nuances. When harvest begins, syrup tends to be clear, and its sweetness is slight. As the season progresses, syrup becomes darker in color and displays distinct aromatic connotations. Indeed, this natural sweetener presents a range of differing aromatic components, including flavors such as vanilla, hazelnut, floral, coffee and spicy aromas.

All the color classes are characterized by a denomination and accompanied by a note about taste [23]:

- Gold (delicate taste);
- Amber (rich taste);
- Dark (strong taste);
- Very dark (robust taste).

All in all, maple syrup quality is driven mainly by its physico-chemical and microbial features. Thus, in order to verify that maple syrup has the appropriate characteristics, namely in terms of color, density and flavor, simple physico-chemical tests are routinely performed. For instance, maple syrup color has been set by measuring the percentage of light transmittance at 560 nm, while sucrose content is determined using a refractometer [24]. These methods are convenient because they provide immediate results [24]. Yet analyses involving more complex techniques that lead to more data, and greater sensitivity, accuracy, and precision in the results are essential to develop processes that allow maple syrup’s functional profile to improve and adulterations and contaminants to be detected. Several studies were conducted to deal with the analysis of the physico-chemical and microbiological parameters of maple syrup. Table 1 presents an overview of the followed analytical techniques and the obtained results.

Maple syrup xylem sap contains naturally occurring molecules and process-derived compounds that are generated during sap evaporation [20]. This means that it contains more than 250 compounds other than sucrose, which is the major maple syrup component [13]. Its minor components include minerals and trace elements, amino acids, other carbohydrates, organic acids, phenolic compounds, sulfur compounds, and pyrazines. Table 2 summarizes the research works carried out as part of a study of maple syrup’s inorganic and organic constituents. Regarding its mineral profile, maple syrup contains considerable amounts of Ca, K and Mg, along with other minerals and trace elements like Zn, P, Mn, Na and Fe [10,25,26,27,28]. Several techniques have been used to determine minerals in maple syrup, including flame and furnace atomic absorption spectroscopy (AAS), inductively coupled plasma-mass spectrometry (ICP-MS) and inductively coupled plasma-atomic emission spectroscopy (ICP-AES). Regarding amino acids, maple syrup is remarkably rich and particular emphasis is placed on D-alanine and other D-amino acids, which have been shown to be generated during the Maillard reaction [29]. By means of a sap samples analysis by metabolomics, the noteworthy work by Garcia et al. [30] reports that amino acid composition varies with season, which is the case of glutamic acid and histidine, whose content is liable to lower as the season progresses, while that of methionine and asparagine tends to grow. The last two have been reported as precursors of the compounds responsible for off-flavors developing in syrup [30]. High-performance liquid chromatography (HPLC) is the gold standard for determining many types of compounds present in maple syrup, namely non-volatile ones, because HPLC allows swift, sensitive, specific, and accurate measurements to be taken, but other equally sound techniques can be used. For example, Pätzold and Brückner [29] employed gas chromatography (GC) coupled with mass detection (MS) to study the amino acids profile of maple syrup. In this case, the polar nature of amino acids required a derivatization step prior to the analysis to make them more volatile and to improve their chromatographic performance. Applying MS is advantageous for its sensitivity and ability to provide structural data [31]. 

Concerning carbohydrates, in addition to sucrose, monosaccharides fructose, and glucose, different oligosaccharides and polysaccharides are found. For instance, Sato et al. [32] developed a method based on hydrophilic interaction chromatography coupled with charged aerosol detection (HILIC-CAD). It allows analyses of up to hepta-saccharides in only 30 min. It enabled the separation and quantification of fructosyl oligosaccharides in maple syrup for the first time. Refractive index (RI) and pulsed amperometric detectors (PAD) are widely used in sugar analyses, and although the RI type is often utilized to analyze known substances, it does not exhibit high sensitivity [32]. PAD yields a high-resolution analysis of multiple sugars [32], but this entails employing an anion exchange column and sodium hydroxide solution as the mobile phase (e.g., [10,33]). Desalting is necessary, which makes identifying new compounds more difficult. So CAD has become increasingly popular [32]. An alternative to chromatographic methods to separate sugars is capillary electrophoresis (CE), which requires a derivatization step to make them electrically charged. This was shown by Taga and Kodama [34]. CE is an appealing option as it incurs moderate operating costs compared to HPLC, employs less solvent and is easily automatable. However, its robustness still raises doubts [35]. To determine organic acids, phenolics and vitamins in maple syrup, HPLC coupled with UV-Vis or diode-array detection (DAD) are some analytical approaches of choice. Compared to UV-Vis detectors, the speed, sensitivity and resolution of DAD are superior despite it being more susceptible to noise interferences [31]. Phenolics are one of the maple syrup constituents to which the most attention has been paid for their numerous health benefits [36,37]. To date, more than 100 phenolics have been identified [13,20,38,39] thanks to the application of techniques such as nuclear magnetic resonance (NMR), which enables the swift analysis of complex mixtures without having to perform separation and/or purification steps, which makes it ideal to analyze maple syrup (e.g., [20]).

One aspect that deserves our attention is that, as the frequency of fraud resulting from admixing inexpensive sugars in maple syrup increases, the development of detection methods is more pressing [40]. Table 3 summarizes the studies performed in this field. Some tools, such as infrared (IR) spectroscopy, are noteworthy. It requires minimal sample pretreatment, is non-destructive [40] and provides reliable results, as shown in the work by Paradkar et al. [41] which successfully reported the addition of beet and cane sugars to maple syrup. Isotope ratio mass spectrometry (IRMS) is another technique with a huge potential for detecting the same type of adulteration, as proved by Tremblay and Paquin [16]. This technique exhibits high precision and the required sample is smaller than that in NMR [40]. 

As a final remark, along with developing analytical tools, the possibility of employing elemental maple syrup content as a strong marker for fingerprinting maple syrup against other syrups must be actively investigated. A literature analysis backs the possibility of identifying percentages by allowing the detection of adulterations to maple syrup with inexpensive syrups employing contents of element. Nevertheless, wide fluctuations in metal contents are reported, which hinders making consistent comparisons, while the possible release of metals from instruments can interfere with acquiring accurate data. These techniques has been used in other foods, such as honey and coffee [42,43]

Jointly, the progress made with new analytical techniques can help with the detection of elemental maple syrup content as a solid marker for fingerprinting this appreciated product, unlike other syrups with lower quality compositions, which should be more exhaustively studied. 

**Table 1:** Physico-chemical and microbiological analyses of maple syrup.

Physico-Chemical and Microbiological Parameters	Studies					
	Samples		Technique	Analysis Details	Main Results	Refs.
	No.	Origin				
Color	33	Canada (Nova Scotia, Quebec, Ontario, New Brunswick) and the United States (New York, Massachusetts, Vermont, New Hampshire)	UV–Vis spectrophotometry	Color intensity was assessed by reading the % light transmittance (%T) at 560 nm.	Color differed markedly depending on the sample origin, with %T ranging from 88.9 to 14.8%. Syrup color darkened near the end of the production season.	[1]
	18 (A) + 7 (NA)	United States (Vermont and New Hampshire)	UV–Vis spectrophotometry	Color measurement was performed using a colorimeter.	The color parameter has not been proved an appropriate tool to distinguish between authentic (A) and non-authentic (NA) maple syrups.	[44]
	233	Canada (Quebec)	UV–Vis spectrophotometry Fluorescence spectroscopy	UV–Vis spectrophotometry: Color intensity was assessed by reading %T at 560 nm. Fluorescence spectroscopy Sample preparation: Syrup samples were diluted with distilled (1:25) prior to analysis.	Intrinsic fluorescence allowed syrup color to be determined with good accuracy (r^2^ = 0.88–0.91).	[45]
	35	Canada (Ontario)	UV–Vis spectrophotometry	Color intensity was assessed by reading %T at 560 nm.	No significant correlation was observed among %T and glucose, fructose or total reducing sugars.	[26]
	20	Canada (Quebec)	UV–Vis spectrophotometry	Color intensity was assessed by reading %T at 560 nm.	Protocatechuic acid and 3-hydroxybenzoic acid concentrations increased with color intensity.	[46]
	32	Canada (state not specified)	UV–Vis spectrophotometry	Sample preparation: Maple syrup samples were diluted 10-fold with water.	Darker-colored syrups displayed stronger antioxidant activity and appeared to contain a higher content of reducing sugars than lighter grades.	[47]
	101	Canada (Quebec)	UV–Vis spectrophotometry Plate count Adenosine triphosphate (ATP) bioluminescence	UV–Vis spectrophotometry: Color intensity was assessed by reading %T at 560 nm. Plate count: Sap samples were diluted in peptone water before being plated on plate count (PC) agar to estimate the total aerobic counts and on acidified potato dextrose (PD) agar to determine fungi. ATP bioluminescence: An assay based on exposing sap samples to the luciferase enzyme and its substrate luciferin was performed.	The ATP bioluminescence measurement of sap allowed a good maple syrup color assessment. In general, lighter syrups were produced from the saps with a low level of microbial contamination, while those with darker colors came from the saps with a high contamination level.	[48]
Density	35	Canada (Ontario)	Gravimetric procedure	Maple syrups were incubated in a water bath at 25 °C and 1 mL was weighed on an analytical balance.	No significant differences were found in density across the maple syrup grades.	[26]
	124	Canada (Quebec)	Refractometry	Total soluble solids–Brix values–were determined using a refractometer.	The total soluble solids values were higher close to the mid-production season.	[49]
	81	Canada (Nova Scotia, New Brunswick, Quebec)	Total solids content	Total solids were determined by evaporating moisture and weighing the dry residue.	No significant differences were found between the different regions of origin of the matrix under study concerning total solids content. This parameter decreased as the production season advanced.	[27]
pH	81	Canada (Nova Scotia, New Brunswick, Quebec)	pH meter		Upon the early production season, maple sap obtained its highest pH value.	[27]
	233	Canada (Quebec)	pH meter Fluorescence spectroscopy		Intrinsic fluorescence provided semi-quantitative information on pH (r^2^ = 0.51–0.75)	[45]
	124	Canada (Quebec)	pH meter		At the beginning of the flow season, maple sap obtained its highest pH value.	[49]
Rheological behaviour	5	Canada (Quebec)	Rheology	The impact of variation in both temperature (5–55 °C) and the soluble sugar concentration (66–75 °Brix) in terms of rheological properties was evaluated.	Maple syrup primarily exhibits Newtonian behavior. Yet, both syrup grade and temperature can impact apparent viscosity within a range from 0.035 to 0.651 Pa s. Furthermore, increasing the maple syrup concentration enhanced its non Newtonian behavior. Overall, the darkest syrups exhibited the greatest viscosity, while very clear ones had the least viscosity.	[50]
	18 (A) + 7 (NA)	Canada (Quebec)	Rheology	A stepped ramp equilibrium flow assay was performed.	Viscosity values ranged between 0.128 and 0.247 Pa s at 25 °C. No relation between maple syrup grade and viscosity was found.	[44]
**Microbiological parameters**						
Bacteria and fungi	233	Canada (Quebec)	Plate count Fluorescence spectroscopy	Plate count: Sap samples were diluted in peptone water before being plated on PC agar to estimate the total bacterial counts and on acidified PD agar to determine fungi. Fluorescence spectroscopy Sample preparation: Syrup samples were diluted using water (1:25) prior to analysis.	Intrinsic fluorescence provided semi-quantitative information on the microbial count (bacterial count: r^2^ = 0.80–0.85; yeasts and moulds count: r^2^ = 0.62–0.73). Fungi and bacteria can be major contributors to maple syrup typicity.	[45]

A—Authentic; NA—Non-authentic (fraudulent or adulterated).

**Table 2:** Chemical analysis of inorganic and organic constituents of maple syrup.

Inorganic Constituents	Studies					
	Samples		Technique	Analysis Details	Main Results	Refs.
	No.	Origin				
Macrominerals and trace elements	2	Canada (Quebec)	Inductively coupled plasma-mass spectrometry (ICP-MS)	Sample preparation: 10 mg of each sample were mixed with nitric acid (1 mL) and digested in a microwave oven. Subsequently, the digested solutions were centrifuged, and an aliquot (200 μL) was further diluted (to 10 mL) with water and analyzed.	The predominant minerals were Ca (212.78–380.48 mg/100 g), K (70.50–128.35 mg/100 g), Mg (48.79–124.51 mg/100 g) and Zn (23.8–90.98 mg/100 g). Determinations were made of the Na (3.52–4.86 mg/100 g), Mn (53.19–58.45 mg/100 g) and Fe (0.44–0.70 mg/100 g) levels.	[25]
	80	Canada (Quebec, Ontario) and the USA (Vermont, Massachusetts, Wisconsin, New Hampshire, Michigan)	Inductively coupled plasma-atomic emission spectroscopy (ICP-AES)	Sample preparation: 0.25 g of sample were diluted with water (25 mL) and directly aspirated into the spectrometer.	The Ca, K and Mg levels respectively ranged between 266 and 1702 mg/L, 1005 and 2990 mg/L, 10 and 380 mg/L. O. The authors indicate that knowledge of maple syrup’s mineral profile might be useful for determining its geographic origin.	[11]
	35	Canada (Ontario)	Inductively coupled plasma-mass spectrometry (ICP-MS)	Sample preparation: 10 mL of nitric acid were added to 5 mL of maple syrup. Then the solution was filtered and placed inside a volumetric flask (50 mL). The final volume was made up with water.	The total mineral content varied between 2988.296–4106.729 mg/L. The Mg, Mn, P, Zn, Ca and K levels ranged between 195.32–238.540 mg/L, 3.107–4.640 mg/L, 2.804–7.731 mg/L, 5.134–6.747 mg/L, 687.310–1554.757 mg/L and 2157.499–2327.860 mg/L, respectively.	[26]
	81	Canada (Nova Scotia, New Brunswick, Quebec)	Flame and furnace atomic absorption spectrophotometry (AAS)	Sample preparation: Syrup samples were diluted (1:50), filtered, and further analyzed.	The Cu, Fe and Zn levels detected in maple syrup samples ranged between 0.09–8.28 µg/mL, 0.41–44.01 µg/mL and 2.81–129.03 µg/mL, respectively.	[27]
	8	Canada (state not specified) and the United States (Vermont, Massachusetts, Maine, New York)	Inductively coupled plasma-mass spectrometry (ICP-MS)	Sample preparation: Digestion using closed-vessel microwave heating.	The Co, Cu, Mn and Zn levels varied between <89 ng/g, <56–2795 ng/g, 1.8–101 µg/g and 5.2–44.2 µg/g, respectively.	[28]
**Organic constituents**						
**Non-volatiles**						
Amino acids	2	Canada (state not specified)	Gas chromatography-mass spectrometry (GC-MS)	Sample preparation: 1 g of sample was diluted with water (5 mL). pH was adjusted to 2.3. Ion exchange solid-phase extraction was applied, followed by drying, redissolving and redrying. Pentafluoropropionic acid anhydride was used as the derivatizing agent. Column: Varian Fused silica capillary column with N-propionyl-L-valine tert-butylamide polysiloxane (Chirasil-Val; 25 m × 0.25 mm; 0.12 µm)	Large amounts of D-amino acids were detected, with D-alanine accounting for 33–34%. D-valine (∼0–4.4%), D-proline (1.8–8.9%), D-serine (∼0–11.3%), D-asx, i.e., a combination of D-aspartate and D-asparagine (3.4–8.0%), D-phenylalanine (6.4–7.0%), D-glx, i.e., combination of D-glutamic acid and D-glutamine (∼0–11.8%) and D-isoleucine (12.7–16.8%), were also detected.	[29]
Carbohydrates	80	Canada (Quebec, Ontario). The USA (Vermont, Massachusetts, Wisconsin, New Hampshire, Michigan)	High-performance anion exchange chromatography coupled with pulsed amperometric detection (HPAEC-PAD)	Sample preparation: For the sucrose analysis, 0.154 g of maple syrup was diluted to 1 L. For the fructose and glucose analysis, 0.875 g of sample were diluted to 250 mL. Column: A Dionex CarboPac PA1 analytical column (250 × 4 mm) Mobile phase: 80 mM sodium hydroxide	The main carbohydrates found were fructose (0.00–3.95%), glucose (0.00–9.59%) and sucrose (51.7–75.6%).	[11]
	1	Canada (state not specified)	High-performance anion exchange chromatography coupled with pulsed amperometric detection (HPAEC-PAD). Gas chromatography-mass detection (GC-MS) Nuclear magnetic resonance (NMR)	Polysaccharides extraction: Performed by ethanol precipitation and dialysis. Polysaccharides purification: Column: A Hiprep Q Sepharose anion exchange column (GE Healthcare Life Sciences; 100 × 16 mm) Mobile phase: Water; sodium chloride Glycosyl composition analysis: Column: Metrohm Hamilton RCX-30 column (250 × 4.6 mm) Mobile phase: Sodium hydroxide Glycosyl linkage analysis: Column: An SP-2330 capillary column (Sigma-Aldrich Supelco; 30 × 0.25 mm)	Isolation of a prebiotic carbohydrate: inulin. Two acidic polysaccharides were identified as arabinogalactans were isolated.	[33]
	4	USA (New Hampshire)	Hydrophilic interaction liquid chromatography coupled with charged aerosol detection (HILIC-CAD)	Sample preparation: Maple syrup was dried, dissolved in water and centrifuged. Then the supernatant was filtered, and acetonitrile was added to adjust the solvent concentration to 75% acetonitrile. Column: Showa Denko Asahipak NH2P-50 4E (4.6 × 250 mm; 5 µm) Mobile phase: Water–acetonitrile	Maple syrup contained not only sucrose, glucose and fructose, but also fructo-oligosaccharides like 1-kestose, blastose, nystose and neokestose, among other unidentified saccharides.	[32]
	1	Canada (Quebec)	Capillary electrophoresis coupled with UV detection (CE-UV)	Sample preparation: Samples were pretreated by enzymatic digestion followed by precolumn derivatisation with 1-phenyl-3-methyl-5-pyrazolone. Column: GL Sciences Fused silica capillary (62 cm; 50 µm) Background electrolyte: 200 mM borate buffer at pH 10.5	9 monosaccharides and 5 disaccharides were detected. Glucose was the major reducing sugar. Mannose was detected at quite high concentrations compared to the other sugars. Arabinose, galactose, N-acetyl-glucosamine, ribose and xylose were also found. Melibiose was detected when samples were treated with invertase, which suggests the presence of raffinose in maple syrup.	[34]
	35	Canada (Ontario)	Enzymatic method	Sample preparation: Samples were diluted (1:25).	Across all the grade syrup samples, no significant differences were observed in glucose (0.670–0.810 g/L), fructose (0.088–0.255 g/L) or in the total reducing sugars (0.870–0.878 g/L).	[26]
Organic acids	80	Canada (Quebec, Ontario). The USA (Vermont, Massachusetts, Wisconsin, New Hampshire, Michigan)	High-performance ion exchange liquid chromatography with UV detection (HPIEC-UV)	Sample preparation: 0.63 g of sample were diluted in 25 mL of water and then filtered. Column: Anion exchange Phenomenex Rezex organic acid column (300 × 7.8 mm) Mobile phase: 0.005 N sulfuric acid	Malic acid (0.1–0.7%) was the main organic acid in maple syrups. Trace levels of citric, fumaric and succinic acids were also detected.	[11]
	2	Canada (Quebec)	High-performance liquid chromatography coupled with diode-array detection (HPLC-DAD)	Column: Restek Allure organic acid column (150 × 4.6 mm) Mobile phase: 100 mM potassium dihydrogen phosphate	Malic acid and fumaric acid levels ranged between 341.85–780.99 mg/100 g and 7.51–15.92 mg/100 g, respectively.	[25]
Phenolics	33	Canada (Ontario)	Modified Fast Blue BB salt method	Experiment procedure: 20 μL of 0.1% Fast Blue BB salt were added to 200 μL of sample. The mix was homogenized and 20 μL of 5% sodium hydroxide were added to each microplate well. Finally, absorbance was measured at 420 nm.	The darker syrups exhibited higher total phenolic contents than the lighter ones; that is, 872.147 μg/mL, 654.780 μg/mL and 415.111 μg/mL for the very dark, dark and amber ones, respectively.	[26]
	5	Canada (Quebec)	High-performance liquid chromatography with UV/Vis spectrophotometric detection (HPLC-UV/Vis)	Sample/extract preparation: Syrup samples (500 mL) were adjusted to pH 7. Three sequential extractions with ethyl acetate were performed. The organic phase that contained phenolics was recovered after all the extractions to be mixed with 100 mL of water to remove residual sugars. The organic phase was dried with anhydrous sodium sulfate and filtered. Finally, the extract was evaporated, dissolved in methanol and dried in nitrogen. Column: Varian analytic column C_18_ (7.8 × 300 mm; 5 µm) Mobile phase: Water/acetonitrile/formic acid (94/5/1, *v*/*v*/*v*)–water/acetonitrile/formic acid (69/30/1, *v*/*v*/*v*)	At the beginning of the season (0%), the total phenolics content in maple syrup was 63.81 g of the gallic acid equivalent (GAE) per 100 g of extract (g GAE/100 g). A significant decline occurred with a value of 17.81 g GAE/100 g up to 75% of the season. At the end of the season (100%), a marked increase took place with 59.41 g GEA/100 g.	[21]
	1	Canada (Ontario)	High-performance liquid chromatography coupled with diode-array detection (HPLC-DAD)	Sample preparation: Maple syrup samples (10 mL) were extracted by liquid-liquid extraction using ethyl acetate 3 times. The combined extracts were evaporated. The residue was dissolved in methanol/water (85:15) and filtered before the HPLC analysis. Column: Agilent Technology Eclipse Plus C_18_ (4.6 × 150 mm; 5 µm) Mobile phase: Methanol/acetonitrile (95:5)–0.05% aqueous formic acid.	The following phenolics were detected and identified from a medium-grade maple syrup: (1) Phenolic acids: (1.1) Benzoic acid and derivatives: (a) gallic acid; (b) 1-O-galloyl-β-D-glucose; (c) protocatechuic acid; (d) gentisic acid; (e) syringic acid; (f) vanillic acid; (g) γ-resorcylic acid); (1.2) Cinnamic acid derivatives: (a) *p*-coumaric acid; (b) 4-methoxycinnamic acid; (c) caffeic acid; (d) chlorogenic acid; (e) ferulic acid; (f) sinapic acid (2) Flavonoids: (a) catechin and (b) epicatechin); (c) kaempferol and its 3-O-β-D-glucoside (d); (e) 3-O-β-D-galactoside; (f) quercetin and its 3-O-β-D-glucoside (g); (h) 3-O-β-L-rhamnoside; (i) 3-O-rhamnoglucoside.	[51]
	1	Canada (Quebec)	High-performance liquid chromatography coupled with UV detection (HPLC-UV) Liquid chromatography coupled with mass spectrometry (LC-MS) Nuclear magnetic resonance (NMR)	Sample/extract preparation: Maple syrup was subjected to liquid-liquid partition with ethyl acetate. It was followed by n-butanol to obtain ethyl acetate and n-butanol extracts, respectively, after solvent evaporation. The butanol extract was further extracted with methanol to yield soluble and insoluble methanol fractions. (1) EtOAc extract: Analytical HPLC: Column: Phenomenex Luna C_18_ column (250 × 4.6 mm; 5 μm) Mobile phase: 0.1% aqueous trifluoroacetic acid–methanol (2) BuOH extract: Analytical HPLC: Column: Phenomenex LunaC_18_ column (250 × 4.6 mm; 5 μm) Mobile phase: 0.1% aqueous trifluoroacetic acid–methanol (2.1) MeOH soluble fraction: Analytical HPLC: Column: Waters Sunfire C_18_ column (250 × 10 mm; 5 μm) Mobile phase: Different methanol/0.1% aqueous trifluoroacetic acid systems	The following phenolics were detected and identified from a very dark-grade maple syrup. (1) Lignans: (a) lyoniresinol ^a^; (b) secoisolariciresinol ^a^; (c) dehydroconiferyl alcohol ^a^; (d) 5′-methoxy-dehydroconiferyl alcohol ^a^; (e) *erythro*-guaiacylglycerol-β-O-4′-coniferyl alcohol ^a^; (f) *erythro*-guaiacylglycerol-β-O-4′-dihydroconiferyl alcohol ^a^; (g) threo-guaiacylglycerol-β-O-4′-dihydroconiferyl alcohol ^b^; (h) *erythro,erythro*)-1-[4-[2-hydroxy-2-(4-hydroxy-3- methoxyphenyl)-1-(hydroxymethyl)ethoxy]-3,5-dimethoxyphenyl]- 1,2,3-propanetriol ^b^; (i) (*erythro,threo*)-1-[4-[2-hydroxy-2-(4-hydroxy-3-methoxyphenyl)-1- (hydroxymethyl)ethoxy]-3,5-dimethoxyphenyl]-1,2,3-propanetriol ^b^; (j) (*threo,erythro*)-1-[4- [(2-hydroxy-2-(4-hydroxy-3-methoxyphenyl)-1-(hydroxymethyl)- ethoxy]-3-methoxyphenyl]-1,2,3-propanetriol ^b^; (k) (*threo,threo*)- 1-[4-[(2-hydroxy-2-(4-hydroxy-3-methoxyphenyl)-1-(hydroxy- methyl)ethoxy]-3-methoxyphenyl]-1,2,3-propanetriol ^b^; (l) *erythro*-1-(4-hydroxy-3-methoxyphenyl)-2-[4-(3-hydroxypropyl)-2,6- dimethoxyphenoxy]-1,3-propanediol ^b^; (m) 2-[4-[2,3-dihydro-3-(hydroxymethyl)-5-(3-hydroxypropyl)-7-methoxy-2- benzofuranyl]-2,6-dimethoxyphenoxy]-1-(4-hydroxy-3- methoxyphenyl)-1,3-propanediol ^b^; (n) acernikol ^b^; (o) leptolepisol D ^b^; (p) buddlenol E ^b^; (q) (1S,2R)-2-[2,6-dimethoxy-4-[(1S,3aR,4S,6aR)-tetrahydro-4- (4-hydroxy-3,5-dimethoxyphenyl)-1H,3H-furo [3,4-c]furan-1-yl]phenoxy]-1- (4-hydroxy-3-methoxyphenyl)-1,3-propanediol ^b^; (r) syringaresinol ^b^; (s) isolariciresinol ^b^; (t) icariside ^b^; (u) sakuraresinol ^b^; (v) [3-[4-[(6-deoxy-α-l-mannopyranosyl)oxy]-3-methoxyphenyl]methyl]-5-(3,4 dimethoxyphenyl)dihydro-3-hydroxy-4-(hydroxymethyl)-2(3H)-furanone ^a^; (w) 5-(3′′,4′′-dimethoxyphenyl)-3-hydroxy-3-(4′-hydroxy-3′-methoxybenzyl)-4-(hydroxymethyl)dihydrofuran-2-one ^b^. (2) Phenylpropanoids: (a) 1,2-diguaiacyl-1,3-propanediol ^b^; (b) 2,3-dihydroxy-1-(3,4-dihydroxyphenyl)-1-propanone ^b^; (c) 2,3-dihydroxy-1-(4-hydroxy-3,5-dimethoxyphenyl)-1-propanone ^b^; (d) 3-hydroxy-1-(4-hydroxy-3,5-dimethoxyphenyl)propan-1-one ^b^; (e) dihydroconiferyl alcohol ^b^. (3) Coumarins: (a) scopoletin ^a^; (b) fraxetin ^a^; (c) isofraxidin ^b^. (4) Stilbene: (a) (E)-3,3′-dimethoxy-4,4′-dihydroxystilbene ^a^. (5) Simple phenolics: (a) 2-hydroxy-3′,4′-dihydroxyacetophenone ^a^; (b) 1-(2,3,4-trihydroxy-5-methylphenyl)ethanone ^a^; (c) 2,4,5-trihydroxyacetophenone ^a^; (d) 3′,4′,5′-trihydroxyacetophenone ^b^; (e) 3,4-dihydroxy-2-methylbenzaldehyde ^b^; (f) catechaldehyde ^a^; (g) vanillin ^a^; (h) syringaldehyde ^a^; (i) gallic acid ^a^; (j) trimethyl gallic acid methyl ester ^a^; (k) protocatechuic acid ^b^; (l) syringic acid ^a^; (m) syringenin ^a^; (n) (E)-coniferol ^a^; (o) tyrosol ^b^; (p) C-veratroylglycol ^a^; (q) catechol ^a^; (r) 4-acetylcatechol ^b^; (s) 4-hydroxycatechol ^b^; (t) 4-(dimethoxymethyl)-pyrocatechol ^b^. (6) Sesquiterpene: (a) phaseic acid ^b^. (7) Non natural phenolic compound: 2,3,3-tri-(3-methoxy-4-hydroxyphenyl)-1-propanol ^ac^ (not originally present in maple sap).	[20,38,39]
Vitamins	2	Canada (Quebec)	High-performance liquid chromatography coupled with diode-array detection (HPLC-DAD)	Column: Phenomenex Luna (250 × 4.6 mm; 5 μm) Mobile phase: 0.1% aqueous trifluoroacetic acid–acetonitrile	The riboflavin and niacin levels fell within the 290.45–410.26 mg/100 g range and the 7.13–9.83 mg/100 g range, respectively.	[25]
**Volatiles**						
Sulphur compounds	4 (Free of BF) + 3 (with BF)	Canada (Quebec)	Headspace solid-phase microextraction combined with gas chromatography-mass spectrometry (HS-SPME-GC-MS)	Sample preparation: A 1 g sample of maple syrup was weighed inside a 10 mL headspace amber vial. 1 mL of sodium chloride 6 M was added. Then the vial was sealed using a screw cap containing a septum. Chromatographic separation: Column: An agilent INNOWax capillary column (30 m × 0.25 mm; 0.25 µm)	Two new volatile sulfur compounds were detected: dimethyl disulfide and dimethyl trisulfide. The first molecule was related to an unpleasant taste in maple syrups.	[52]
Pyrazines	3	Canada (Quebec)	Gas-liquid chromatography (GLC)	Sample preparation: 100 g of sample were diluted with water (100 mL). pH was adjusted to 3. Then 30 mg of sodium chloride were added. The mixture was extracted with diethyl ether. This was subsequently followed by separating the aqueous phase, adjusting to pH 11 and extracting with dichloromethane. Finally, the extract was concentrated. Column: Supelcowax 10^TM^ fused silica (30 m × 0.32 mm; 0.1 µm)	The total pyrazine content in different maple syrup classes differed markedly, with “medium” grade maple syrups exhibiting the highest contents (68 ng/g), whilst “amber” grade syrups showed the lowest levels (48.89 ng/g).	[53]
	4	Canada (Quebec)	Headspace solid-phase microextraction combined with gas chromatography-mass spectrometry (HS-SPME-GC-MS)	Sample preparation: A 1 g maple syrup sample was weighed inside a 10 mL headspace vial. 1 mL of sodium chloride 6 M was added. The vial was sealed using a screw cap containing a septum. Chromatographic separation: Columns: Supelco Supelcowax 10 and Varian VF-5ms (both with 30 m × 0.25 mm; 0.25 µm).	27 pyrazines were identified, of which 15 were reported as flavor components. All the molecules were alkylpyrazines (except 2-methoxy-3-(1-methylethyl) pyrazine and tetramethylpyrazine).	[54]

BF—Buddy off-flavor; ^a^—Compounds detected in the BuOH extract; ^b^—Additional compounds detected in the EtOAc extract; ^c^—Additional compound detected in the MeOH soluble faction of the BuOH extract.

**Table 3:** Detection of adulterants in maple syrups.

Adulterants	Studies					
	Samples		Technique	Analysis Details	Main Results	Refs.
	No.	Origin				
Beet and cane sugar	4 (A) + 10 (NA)	Canada (Quebec). The USA (Vermont)	Site-specific deuterium nuclear magnetic resonance (SNIF-NMR)	Sample preparation: Maple syrup samples were diluted with water and underwent a fermentation process. Ethanol was distilled and its extractive yield was calculated. Analysis: The ethanol analysis was performed by a high-field NMR spectrometer.	The (D/H)_I_ value of the exogenous sugars-adulterated samples considerably differed from that of the pure maple syrups. More specifically, the (D/H)_I_ parameter decreased when adding beet sugar to maple syrup at 1 mg/g for every 10% added sugar. It grew when cane sugar was added, specifically at 1 mg/g per 10% added sugar. One disadvantage of this method is that when it is applied to specific sugar beet, cane or corn mixtures, its sensitivity decreases, but this may be useful for combining it with the ^13^C ratio measurement.	[55]
Beet and cane invert syrups Beet and cane sugar solutions	1 (A) + 4 (NA)	Canada (state not specified)	Fourier transform infrared (FTIR) and near-infrared (NIR) spectroscopy conjugated with a discriminant analysis, i.e., a canonical variate analysis (CVA), a linear discriminant analysis (LDA) and a quantitative analysis, i.e., partial least squares (PLS) and principal component regression analysis (PCRA)	Sample preparation: Pure maple syrup was adulterated with varying amounts of cane and beet invert syrups, and also with 60% beet and cane sugar solutions.	All in all, it is possible to detect adulterants like pure beet and cane sugar solutions by both NIR and FTIR, but FTIR outperforms NIR in detecting invert syrups.	[41]
Beet and cane sugar	231 (A) + 112 (NA)	Canada (Quebec). The USA (Vermont, Maine, New Hampshire, New York)	Stable carbon isotope ratio mass spectrometry (δ1^3^C IRMS)	Malic acid, an organic acid that is naturally present in maple syrups, was suggested to act as an internal isotopic standard to improve the adulteration LoDs. Sample preparation/malic acid isolation: Organic acids were isolated from maple syrup by lead precipitation. Malic acid was separated by means of preparative reversed-phase liquid chromatography. Column: Phenomenex Maxcil C_18_ analytical column (250 × 21.21 mm; 5 µm); Mobile phase: Potassium dihydrogen phosphate adjusted to pH 2.4	The mean δ^13^C of the maple syrup sample sugars was −24.07‰, while that of malic acid was −26.71‰, which agree with the stable carbon isotopic ratios characteristic of C_3_ plants. The correlation between sugars and malic acid was good, i.e., r = 0.34. This proves that malic acid is an appropriate internal standard. A new calculation method was developed and applied to improve the decision limit of maple syrup adulteration according to the correlation between the δ^13^C_malic acid_ and the δ^13^C_sugars_-δ^13^C_malic acid_ (r = 0.704). The theoretical LoD markedly lowered when this technique was applied compared to the usual two standard deviation (SD) method, particularly for the beet sugar-adulterated maple syrup (24 ± 12% vs. 48 ± 20%).	[16]
Cellulose gum	18 (A) + 7 (NA)	The USA (Vermont, New Hampshire)	Rheology	Viscosity analysis: A stepped ramp equilibrium flow test was performed. Studying the impact of cellulose gum on syrup rheological behavior: An oscillatory frequency sweep test was run.	The dynamic rheological method applied detected, with adequate sensitivity, changes in viscosity caused by the addition of polymers such as cellulose gum, wherefore this technique can be successfully employed to detect this type of adulteration.	[44]

A—Authentic; NA—Non-authentic (fraudulent or adulterated); LoD—Limit of detection.

## 3. Nutritional Profile and Health Impacts

Boiling maple sap concentrates carbohydrates and other elements, which is how maple syrup is made. The timing of collection affects the amounts of micronutrients, macronutrients and phenolics in maple sap, which result in variations in maple syrup [56]. Maple syrup is high in phytochemicals, macronutrients (sucrose) and micronutrients [7,11,57], and sucrose is its main component (96%), with a small amount of hexoses. Much lower concentrations of minerals, trace elements, organic acids, phytochemicals (lignan, stilbene, coumarin, phenolic compounds) and vitamins are found than in sugars [8,11,18,20,38,39,51,58]. St Pierre et al. [7] compared chemical maple syrup components to those of other natural sweeteners, including honey, molasses, blue agave syrup, brown rice syrup and golden corn syrup (abscisic acid [ABA], carbohydrates and phenolics). This research concluded that when compared to brown rice syrup, corn syrup, and pure dextrose, maple syrup significantly reduced the peak and total responses of glucose, insulin, amylin, and gastric inhibitory polypeptide (GIP). Molasses and agave syrup both had similar metabolic effects to maple syrup, however, honey increased the peak responses of insulin, amylin, and GIP. The elemental composition of maple syrup and the metabolic reactions to it in rats suggest that it is a healthy natural substitute for refined sugar.

Multiple bioactive compounds appear to be involved in the distinct flavor of maple syrup, such as carbonyl compounds [59,60], phenolics [61,62,63], pyrazines, alcohols, acids, and furan derivatives [53,54,59,60,64,65,66].

Apart from process-generated components, maple syrup often contains phytonutrients obtained from sap [8,11,20]. Maple syrup has been found to include vanillin, coniferyl alcohol, protocatechuic acid and syringic aldehyde [1]. Syrup extracts contain coniferaldehyde, vanillin, syringaldehyde, benzoic acid derivatives, cinnamic acid and flavonoids (flavonols) [63].

Thériault et al. [21], Legault et al. [67], Kamei et al. [68], Maisuria et al. [69] and Liu et al. [70] have found that maple sap and the phenolic-rich extracts of maple syrup perform antiproliferative, antiradical, antimicrobial, antimutagenic and antioxidant activities. From maple sap and syrup, Thériault et al. [21] identified aglycone phenolic and glycosylated molecules. Glycosylated sap/syrup components have stronger antioxidant and antiradical properties than aglycones. SOS induction suppression in *Salmonella typhimurium* TA1535/pSK1002 that contained fusion gene umuC-lacZ was used to investigate each chemical’s antimutagenic activity. Glycosylated phenolic compounds’ antimutagenic properties are optimal for 25% of the season for syrup and for 75% of the season for sap at different times of the year. Aglycones in sap present the greatest antimutagenic feature for 75% of the season, whereas aglycones in syrup do so for 25–100% of the season.

Li and Seeram [20] applied the DPPH (2,2-diphenyl-1-picrylhydrazyl) experiment to separate phenolics from MS-BuOH and to compare their antioxidant activities to a positive antioxidant control (butylated hydroxytoluene). Coumarins have stronger antioxidant capacity than stilbenes and lignans among isolated phenolics. Zhang et al. [25] investigated the biological activity and safety characteristics of maple syrup extract. In vitro, the extract demonstrated anti-inflammatory and antioxidant (DPPH test) properties, and inhibited glucose intake (by HepG2 cells). The study by Liu et al. [70] found that phenolics-enriched maple syrup extract (61.7 g/mL) scavenged ∼50% DPPH and decreased free radical production by 20% throughout the glycation process.

The biological effects of an organic phenolics-rich, sugar-reduced maple syrup extract employed as a new dietary component high in phenolics were studied by Nahar et al. [58]. With a lipopolysaccharide-stimulated RAW 264.7 murine macrophage cell model, anti-inflammatory MS-EtOAc activity and its purified isolates were investigated. MS-EtOAc reduced nitric oxide (NO) and prostaglandin E2 (PGE2) generation by lowering NO synthase (iNOS) levels, while upregulating the protein expression of cyclooxygenase 2 (COX-2) mRNA. The most effective inhibitor of PGE2 and NO was (E)-3,3′- Dimethoxy- 4,4′- dihydroxystilbene. In a mouse model of Alzheimer’s disease, a phenolics-enriched maple syrup extract presented anti-neuroinflammatory actions [71]. The expression of several inflammatory proteins, including Alzheimer’s disease risk-associated proteins, was reduced by maple syrup extract. The impact that maple syrup extract had on hepatic gene expression in mice on a high-fat diet has been reported by Kamei et al. [68] and Kamei et al. [72]. According to changes in the expression of the genes associated with lipid metabolism and immune response, maple syrup extract can help to attenuate hepatic inflammation in mice.

The antiproliferative effects of botj MS-EtOAc (Grades C and D) extracts and purified phenolics against non-tumorigenic (CCD-18Co) and human tumorigenic (HCT-116, HT-29, CaCo-2) colon cells have been investigated by González-Sarrías et al. [5]. Extracts MS-EtOAc, MS-BuOH and MS-MeOH proved more effective against tumorigenic colon cells than non-tumorigenic colon cells. The most active compounds were gallic acid, syringaldehyde, catechaldehyde and catechol, whose presence in Grade D MS-BuOH extract could explain its anticancer properties. Cancer apoptosis was not caused by extracts, but they did cause cell cycle arrest. The synergistic action of various phenolics might explain the high activity of MS-BuOH extract. González-Sarrías et al. [73] studied the anti-proliferative effects of ginnalins A-C on tumorigenic and non-tumorigenic colon (HCT-116) and breast (MCF-7) cells. Ginnalins A-C were twice as active against tumorigenic vs. non tumorigenic cells. This finding indicates that their selectivity for cancer cells is stronger. Ginnalin A was more active than ginnalins B and C. Maple phenolics may have a cancer chemopreventive effect via cell cycle arrest, as well as their direct cytotoxic effect. Yamamoto et al. [74] investigated the effects of dark-colored maple as a drug for gastrointestinal cancer therapy. It suppressed protein kinase B phosphorylation and further decreased cell proliferation by limiting protein kinase B activation. According to Yamamoto et al. [75], MS-EtOAc reduced cell proliferation, migration, and invasion in pancreatic cancer cells.

St-Pierre et al. [7] found several maple syrup components with health-promoting effects on glucose homeostasis. α-glucosidase inhibitory activity has been found in maple syrup phenolics [7,20,76]. ABA is a phytohormone in maple with promising anti-diabetic properties [7,77,78,79,80]. Furthermore, ABA has been shown to protect against Type-2 diabetes [78,80,81]. The impact of MS-EtOAc and MS-BuOH extracts on inhibiting carbohydrates by hydrolyzing enzymes (i.e., α-glucosidase) has been studied by Apostolidis et al. [82], where MS-BuOH exhibited more marked inhibitory action than EtOAc and was posed as a potential adjuvant with antihyperglycemics for Type-2 diabetes management. How phenolics-rich maple syrup extract affects Type-2 diabetes model mice has been evaluated by Toyoda et al. [81]. In Type-2 diabetic mice livers, treating rats with maple syrup extract inhibited fat accumulation by down and upregulating lipolysis hepatic enzymes and lipogenesis. The same research group [83] revealed how maple syrup extract can help with some dyslipidemia symptoms by another experiment.

In healthy rats, St-Pierre et al. [7] examined maple syrup metabolic reactions against other sweeteners. Dextrose corn syrup and brown rice syrup resulted in lower peak responses insulin, glucose, amylin, and gastric inhibitory polypeptide than maple syrup. Maple syrup’s unique properties, plus metabolic reactions to its consumption in animals, indicate that it might be a healthy alternative to other sugars.

Dupuy and Tremblay [84] examined the effects that maple-sweetened beverages (sap or syrup) have on cognitive flexibility while practicing high-intensity exercise using a commercial sports drink, water, and glucose. The glycemic index of maple products was lower than the glycemic index of glucose alone.

Antimicrobial potential and a significant synergistic effect with various antibiotics were found in a phenolics-rich maple syrup extract against Gram-negative microorganisms (*Escherichia coli*, *Proteus mirabilis* and *Pseudomonas aeruginosa*). Catechol was effectively combined with antibiotics and other phenolics in a phenolics-rich maple syrup extract to inhibit microbial growth [70].

In summary, we would like to remark that, in addition to the main constituent sucrose, maple products also include phenolics, pyrazines, vitamins, minerals, organic acids, and phytohormones. These bioactive substances have the potential to be valuable due to their positive impacts on health, such as their antioxidant, antiproliferative, and antimutagenic properties. It is proposed that quebecol, lariciresinol, and secoisolariciresinol serve as distinctive markers for maple products and are uncommon in syrups made from other plants [56].

## 4. Safety and Quality Control

### 4.1. Food Safety

Food safety is a key determinant in the quality of maple syrup, wherefore aspects related to it should be seriously considered. In particular, the risk of contamination with metals and toxic microorganisms, namely fungi [49,85] given the potential for occurrence of mycotoxins even at low water activity levels (aw) [86], which still needs to be investigated. Table 4 summarizes the main findings in terms of the occurrence of contaminants in maple syrup.

### 4.2. Quality Control

**Figure 1:**
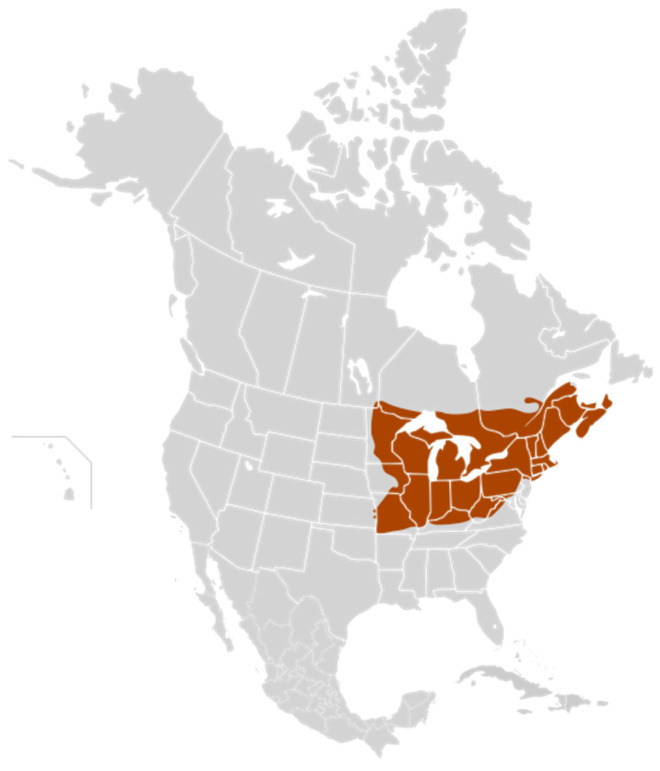
Regions of maple syrup production in Canada and the USA according to the Maple Syrup Producers’ Association of Ontario [94].

As in other natural sweeteners obtained from vegetables, maple syrup is collected from maple sap trees in some regions of eastern Canada and northwestern USA (Figure 1). It being a seasonal product with given harvest dates means that it can be collected in about 35 days. Its characteristics depend on the production region where it is harvested, and it is affected by both the weather and its extraction process (boiling), performed to obtain the final syrup. For these reasons, Canadian and US maple syrup production can fluctuate yearly due to changes in the weather. The principal Canadian maple syrup production concentrates in the eastern province of Quebec. Therefore, Quebec is the leading maple syrup producer in Canada, and has by far the most maple farms, taps and, as a result, the most maple syrup [87].

Quebec is the province with the highest maple syrup production levels in Canada, which leaves the second highest producing province, New Brunswick, far behind (Table 5). New Brunswick’s production levels are the same as that of Vermont in the USA, which is by far the nation’s biggest maple syrup producer, followed by New York (Table 5).

Although there are only four species of maple trees used to collect maple sap and to obtain maple syrup, there are actually over 150 maple tree species worldwide [88]. The more important producing species are *Acer saccharum* (70%) and *Acer rubrum* (29%). Nevertheless, other silver species, such as silver maple *Acer saccharinum* and black maple *Acer nigrum* (1%), can be considered as maple sap-producing species.

We briefly summarize the maple sap-producing description. Maple sap is normally collected in February-March when weather conditions make it easy to collect sap. This is done by boring holes in maple tree trunks. The encrusted tap permits sap to flow by pipelines to buckets. Sap is then concentrated by boiling it down into maple syrup [89]. For Canada, maple sup products (sugar and maple butter, maple syrup and taffy) are very interesting in economic and cultural terms because maple product exports have constantly increased and exported maple products now amount to more than 385 million Canadian dollars.

On the other hand, factors such as processing procedures, geographical and seasonal fluctuations, and microbiological contamination can impact maple syrup composition. The seasonal compositional changes in Nova Scotia syrup minerals are reported by Nimalaratne et al. [1], with K (2431–2547 mg/L), Ca (568–900 mg/L) and Mg (120–158 mg/L) being the main minerals, followed by Mn (15–20 mg/L), P (7–13.5 mg/L) and Zn (2.8–3.8 mg/L). Brix (61.6–70.2°) pH, color, and mineral content (2.6–4.8 g/L) vary depending on origin, but pH, sugars and Brix do not alter across seasons.

#### 4.2.1. The Maple Syrup Quality Standard

Maple trees accumulate starch as they grow, which is converted into sugar during spring thaws and mixes with water absorbed by tree roots to create maple sap, which generally flows between February and April every year [90]. Producers employ tubing systems, RO, and high-performance evaporators to collect sap before boiling it down to obtain maple syrup. Canadian maple syrup products range from traditional maple syrup to maple butter, maple candy and maple sugar, plus a wide range of maple syrup-containing products.

Canadian maple syrup takes two grade names: “Canada Grade A” (further graded into four color classes– “Golden, Delicate Taste”, “Amber, Rich Taste”, “Dark, Robust Taste” and “Very Dark, Strong Taste”–that typically reach consumers and commercial markets); and “Canada Processing Grade”, which has no color classes and is frequently applied to large-scale commercial applications [22]. In 2020, they harvested 13.2 million gallons. Thanks to favorable spring weather and more taps, higher yields account for more production. Prices in other maple-producing provinces are set by producers. As a result, they can substantially vary. Prices in Quebec are controlled by the Régie des Marchés Agricoles et Agroalimentaires du Québec. This organization helps to stabilize prices from year to year. In 2020, the price in Quebec remained at $38.55/gallon, and the total maple products value was $509.2 million [90]. Maintaining final product quality is most important. Thus, for economic performance to improve, the increase in maple farmers and maple taps denotes the profitability of such activities [68]. There are several quality guidelines for the production and commercialization of maple syrup from Canada and the US [91,92], but they are all generic guidelines for food safety without a focus on specific hazards.

#### 4.2.2. Factors That Can Influence Final Maple Syrup Composition

Unlike other sugar sources, maple syrup has a unique characteristic flavor that depends on its composition, e.g., organic compounds (sugars, alcohols, ketones, aldehydes), micronutrients and phytochemicals, of which more than 200 compounds appear in maple syrup that are either natural or are transformed during processing [13]. Hence this unique composition can be used for either detecting possible fraud and adulteration with other syrups of lesser quality, such as cane sugar, beet, and corn [13], or for confirming those classified according to strict Canadian and US regulations.

Thus, many essential and non-essential metals are present in maple sap, and their numbers may rise during maple sap concentration done by boiling [13]. Several studies have shown the utility of determining some essential elements, such as salt content, to be used in maple syrup characterization and for differentiating it from other syrups depending on the relation of these compounds.

Several authors, such as Lagacé et al. [49], have studied maple sap during different harvest periods to show variations in its organic composition: sugar (sucrose, fructose, and glucose), organics acid, and phenolic compounds. This means having to change some intrinsic factors, such as maple tree sap flowing in trunks. Moreover, other changes could modify its composition given the microbial maple sap population (fungi and total aerobics), which progressively increase during the season [9,93]. After maple sap is collected, it is contaminated by microorganisms, which are responsible for sucrose hydrolysis and the final presence of fructose and glucose in maple syrup [50].

## 5. Applications in the Food Industry and Sustainability Issues

Maple syrup comprises sugar, trace amounts of organic acids, free amino acids, protein, minerals, and phenolic compounds [11]. These trace components allow maple syrup’s taste profile to be distinguished from that of sucrose. They are what contributes to its potential health benefits when compared to sucrose [96]. Maple syrup can be manufactured from a combination of corn syrup, maple coloring and flavoring. However, maple syrup in its natural state contains minerals like calcium and potassium, which may not be at the same levels when it is manufactured [97]. Pure maple syrup possesses specific standards for clarity, density, flavor, and color properties, along with descriptors that typically include woody, vanilla, caramel, floral, fruit and herbaceous [11,19].

When considering maple syrup applications as an ingredient in the food industry, the chemical analysis and nutritional profile of maple syrup are essential, as discussed in the previous Section 2 and Section 3. A better understanding of the physico-chemical and microbiological analyses, organic and inorganic constituents, adulterants, and contaminants of maple syrup will help to ensure its uniformity and quality standards in the food industry.

According to the International Maple Syrup Institute, pure maple syrup has a small market share in the USA, Canada and elsewhere overseas. For example, in the USA, maple syrup, along with honey, represents 1% of all the sweeteners delivered for food and beverage uses [98]. Maple syrup is not only employed as a pancake topping, but its unique characteristics are mediated by bioactive compounds, which makes its suitable for several culinary and industrial applications.

Several other maple syrup applications are found in the food and beverages industry and have been paid some attention as part of the culinary education guide compiled by Kimball [99]. They include the following:

- Maple butter: It is thick, but spreadable, and is also called maple cream or spread. It is a whipped version of pure maple syrup;
- Clear maple: It begins as maple syrup. It is then altered by adding a processing aid, which is removed later to create higher invert sugar content. The resulting product is a product with a honey-like consistency made from pure maple syrup;
- Pure maple syrup concentrate: It is produced after removing almost 50% of the sucrose content in pure maple syrup;
- (Medium or coarse) maple flakes: They are made with pure maple syrup that has been dehydrated by means of a unique exclusive process;
- Maple jelly: It is made from pure maple syrup with a jelly-like structure and can be used for culinary purposes.
- Maple sugar: It is made from pure maple syrup by dehydration into granulated sugar crystals. It can be replaced at 1:1 with regular granulated sugar in the majority of formulae and recipes;
- Maple vinegar: It is produced from pure maple syrup by alcoholic fermentation and acetic fermentation processes. Adding maple vinegar creates a signature salad dressing.

Maple syrup can be employed in diverse menu items to sweeten tea, lemonade, regular coffee, and café lattes. Acorn squash or sweet potatoes can be glazed with maple syrup. Maple can be used to create a sweet and savory barbecue sauce, and drizzled on pears, walnuts, and gorgonzola pizzas. Baked maple-kissed goods, ice-cream and desserts can be prepared to gain a better taste. For a wider application, there is a need to utilize maple syrup on an industrial scale that will ensure that more consumers gain from its naturalness and health benefits.

Maple syrup processing mainly involves water removal to increase its viscosity. The main industrial processing techniques are based on conventional evaporation and reverse osmosis (RO), as described by Ramadan et al. [56].

**Figure 2:**
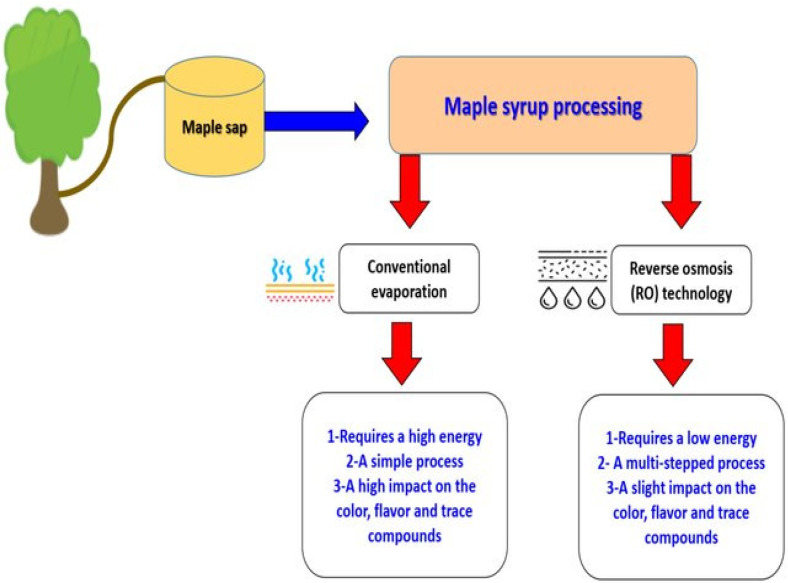
A comparison of the two processing methods to produce maple syrup (Adapted from Ramadan et al. [56]).

The two processing methods shown in Figure 2 will have impacts on the quality characteristics of the obtained maple syrup. When comparing the two processes, evaporation can result in varying sensory attributes, such as color and flavor, while RO performed at room temperature does not change maple syrup’s chemical properties [56]. The heating and evaporation steps of maple sap are critical maple syrup processing stages. Flavor and color are essential factors that affect the maple syrup grade, which range from very dark-colored strong-flavored syrup to very light-colored delicate-flavored syrup [11]. Maple syrup flavor is also influenced by the regions where sugar maple trees grow. The amount of nitrogen compounds, phenolic compounds, flavonoids, and organic acids in maple sap may vary throughout the maple syrup season, and also from one season to the next, according to the region, and even from one maple tree to the next [99].

When comparing RO to conventional evaporation, which requires high energy use, RO can be used to concentrate maple sap to cut energy costs [100]. In industry, most RO techniques are extensive processes involving many steps [101]. To obtain 1 kg of maple syrup (68% sugar), 34 kg of maple sap (2% sugar) are required, plus conventional evaporation. Conversely, when RO concentrates maple saps up to 20%, only 3.5 kg of sap are necessary to obtain 1 kg of maple syrup [102].

It is crucially important to note that quality standards of the obtained maple syrup products are affected by the processing parameters; the phytochemical profile of these products will also influence flavor and color when they are considered for applications in the food industry. Recently, it was suggested that quebecol, lariciresinol, and secoisolariciresinol are distinct markers for maple products since they are not common in other plant-derived syrups [56]. Another significant limitation in the quest for further industrial application of maple syrup is microbial contamination of the maple sap which will affect maple product quality. The application of a continuous heat treatment on buddy syrups for 2 h at 104.5 °C was able to remove the buddy off-flavor by reducing the volatile dimethyl disulfide content in maple syrup which is responsible for this off flavor [52]. Henceforth, it is important to conduct further research on how processing techniques and environmental conditions affect the phytochemicals profile and biological effects of industrially produced maple food products.

With increasing climate change awareness, there is contention about evidence for a climate optimum for syrup production based on a standardized protocol for collecting sap from individual trees under natural conditions. There are indications that there will be shorter sap flow with warming winter temperatures if traditional tapping schedules are maintained [103]. Modeling the relations among climate, sap flow and sugar concentration is necessary to gain an understanding of the basic eco-physiological responses underlying climate effects on syrup production [104]. As Duchesne and Houle [105], Collins et al. [106], and Bal et al. [107] misrepresented this, further research is necessary [104]. In Canada, producers must adhere to the strict standards and guidelines set by Canadian Law and the Federation throughout the maple syrup production process. It is important that the maple syrup value chain is sustainable. The role of maple syrup as a non-timber forest product, an alternative to extractive forest timber activities within the community will contribute to subsistence needs and help diversify and supplement rural incomes [108]. Generally, harvesting is carried out in such a way that sugar maple trees are tapped in a different area from that of the year before to preserve tree health. For instance, the Canadian “Preservation of Agricultural Land and Agriculture Activities Act” forbids felling a whole maple tree in an agricultural area [57]. While maple syrup demand as a natural and healthy sweetener alternative in the food industry increases, the entire value chain’s sustainability is an important criterion from production to consumption.

## 6. Conclusions

This review explores the potential of maple syrup as a natural sweetener to be used in human diet. As consumers are showing considerable interest in demanding more natural ingredients in their food items, it critically examines maple syrup, along with its quality characteristics, and nutritional and health impacts. In fact, current scientific evidence indicates that phenolic compounds play a key role in the body’s defense, protecting it from damage caused by reactive oxygen species known to be involved in the genesis of various pathologies, cardiovascular, oncological, autoimmune, degenerative, etc. That said, the potential of maple syrup, derived from *Acer saccharum* Marsh., as a source of nutrients and bioactive compounds is immense and deserves to be highlighted.

Therefore, the objective of this paper was to perform a global review on maple syrup as an interesting sweetener with application in the food sector. Furthermore, this review also aims to contribute to the improvement of food availability in a sustainable way and to provide also economic welfare. Finally, this sweetener can offer an important contribution for the development of new food products in the future and can contribute to decisive improvements in public health.

## Figures and Tables

**Table 4:** Detection of contaminants in maple syrups.

Contaminants	Studies					
	Samples		Technique	Analysis Details	Main Results	Refs.
	No.	Origin				
Toxic metals	81	Canada (Nova Scotia, New Brunswick, Quebec)	Furnace atomic absorption spectrophotometry (FAAS)	Sample preparation: Syrup samples were diluted (1:50) and filtered prior to the analysis.	The Pb levels detected in the maple syrup samples ranged between 0.33 and 2.68 µg/mL.	[27]
	2	Canada (Quebec)	Inductively coupled plasma-mass spectrometry (ICP-MS)	Sample preparation: 10 mg of each sample were mixed with nitric acid, and digested in a microwave oven. Subsequently, the digested solutions were centrifuged. A 200 μL aliquot was further diluted to 10 mL with deionized water and analyzed.	Al levels were between 0.32 and 0.46 mg/100 g.	[25]
	8	Canada (state not specified). The USA (Vermont, Massachusetts, Maine, New York)	Inductively coupled plasma-mass spectrometry (ICP-MS)	Sample preparation: Digestion by closed-vessel microwave heating.	As, Cd, Pb and V levels varied between 1.3–7.1 ng/g, 1.5–49 ng/g, 18–367 ng/g and <1.1 ^a^–187 ng/g, respectively.	[28]
Microorganisms	101	Canada (Quebec)	Plate count Adenosine triphosphate (ATP) bioluminescence	ATP bioluminescence: An assay based on exposing maple samples to the luciferase enzyme and its substrate luciferin was performed.	The maple syrups produced from the sap with higher ATP bioluminescence values were darker and had unpleasant flavors.	[48]
	183	Canada (Ontario)	Plate count Polymerase chain reaction (PCR)	Microbiological analysis: Syrup samples were inoculated in two media: Yeast Extract Sucrose (YES) agar and Dichloran Glycerol (DG18) agar. Distinct colonies were placed on 2% Malt Extract (ME) agar for further DNA extraction, and on ME and Czapek Yeast Extract (CYE) agars for identification purposes.	*Eurotium herbariorum* was the most prevalent fungus found in the maple syrup samples. It was followed by *Penicillium chrysogenum*, three *Aspergillus* species (*A. penicillioides*, *A. restrictus*, *A. versicolor*) and two *Wallemia* species (*W. muriae* and *W. sebi)*. *Cladosporium cladosporioides* was also isolated.	[85]

^a^—LoD for V = 1.1 ng/g, LoD—Limit of detection.

**Table 5:** Maple syrup production [95] per province (in thousands of gallons).

	Maple Sap Production							
Country	2015	2016	2017	2018	2019	2020	2021	2022
**Canada**	8908	12,160	12,512	9796	13,204	14,294	11,311	
Quebec	8090	11,185	11,493	8914	12,033	13,210	10,027	15,949
New Brunswick	430	528	551	361	598	561	786	
Ontario	369	398	425	465	502	467	462	
**USA**	3204	4207	4271	4159	4240	4111	3721	5028
Vermont	1410	1990	1980	1940	2070	1950	1750	2550
New York	601	707	760	806	820	804	647	845

(1) Conversion factors: 1 gallon of syrup equals 10.0 pounds of maple sugar. One gallon of syrup weighs 13.24760 pounds. (2) 1 gallon US: 3.785 L.

## Data Availability

Not applicable.
